# Method for mapping population-based case-control studies: an application using generalized additive models

**DOI:** 10.1186/1476-072X-5-26

**Published:** 2006-06-09

**Authors:** Thomas Webster, Verónica Vieira, Janice Weinberg, Ann Aschengrau

**Affiliations:** 1 Department of Environmental Health, Boston University School of Public Health, Talbot 2E, 715 Albany Street, Boston, MA 02118, USA; 2 Department of Biostatistics, Boston University School of Public Health, Talbot 2E, 715 Albany Street, Boston, MA 02118, USA; 3 Department of Epidemiology, Boston University School of Public Health, Talbot 2E, 715 Albany Street, Boston, MA 02118, USA

## Abstract

**Background:**

Mapping spatial distributions of disease occurrence and risk can serve as a useful tool for identifying exposures of public health concern. Disease registry data are often mapped by town or county of diagnosis and contain limited data on covariates. These maps often possess poor spatial resolution, the potential for spatial confounding, and the inability to consider latency. Population-based case-control studies can provide detailed information on residential history and covariates.

**Results:**

Generalized additive models (GAMs) provide a useful framework for mapping point-based epidemiologic data. Smoothing on location while controlling for covariates produces adjusted maps. We generate maps of odds ratios using the entire study area as a reference. We smooth using a locally weighted regression smoother (loess), a method that combines the advantages of nearest neighbor and kernel methods. We choose an optimal degree of smoothing by minimizing Akaike's Information Criterion. We use a deviance-based test to assess the overall importance of location in the model and pointwise permutation tests to locate regions of significantly increased or decreased risk. The method is illustrated with synthetic data and data from a population-based case-control study, using S-Plus and ArcView software.

**Conclusion:**

Our goal is to develop practical methods for mapping population-based case-control and cohort studies. The method described here performs well for our synthetic data, reproducing important features of the data and adequately controlling the covariate. When applied to the population-based case-control data set, the method suggests spatial confounding and identifies statistically significant areas of increased and decreased odds ratios.

## Background

Mapping spatial distributions of disease occurrence can serve as a useful tool for identifying exposures of public health concern, e.g., [1]. Epidemiologists often produce disease maps by combining registry information with census data, plotting mortality, incidence or prevalence by town, census tract, or other geographical division. While such maps can provide etiologic clues, they have important limitations. Registries usually collect information on only a few common covariates such as age and gender, potentially causing spatial confounding. For example, a local increase in lung cancer incidence could be due to spatial clustering of smokers. Smoking is an important risk factor for lung cancer that cannot be controlled in standard maps because the data are often not routinely collected. Registries typically record residence at time of diagnosis. For outcomes with long latencies, important exposures may have occurred many years before at different locations. Maps that ignore latency may tend to be flatter if population movement is random with respect to disease status [2]. Census data are typically aggregated within arbitrary geographic units (e.g., towns), producing poor spatial resolution, and large between-area differences in precision. The choice of areal units employed for mapping can significantly affect the resulting map [3].

These mapping issues can be addressed using the much richer data obtainable from population-based case-control and cohort studies. Using a disease registry, cases in a given geographic area can be identified. The population giving rise to the cases must also be enumerated or sampled. When controls are appropriately sampled from the population giving rise to the cases, the case-control ratio (disease odds) in a subset of the area should be proportional to the disease incidence rate [4]. While expensive and time-consuming, population-based case-control and cohort studies can collect detailed information on residential history and a large number of potential risk factors. The covariate information collectable from such studies permits much better control of confounding than data routinely collected by registries. Considering residence as a proxy for exposure, epidemiologists can account for latency by mapping where people lived for specified lengths of time before they were diagnosed (With only residence at diagnosis, one can at best analyze rates at various time points in the past, a quite different kind of map). Geocoding the residential locations of cases and controls produces point-based data, avoiding aggregation by arbitrary geographical units.

Our goal is to develop practical methods for epidemiologists using readily available software. This paper will describe the statistical, mapping, and epidemiological methods we employ to map case-control data. We provide examples using both synthetic data and real data from a population-based case-control study of breast cancer on Upper Cape Cod, Massachusetts, USA.

## Results

### Synthetic data

**Figure 1:**
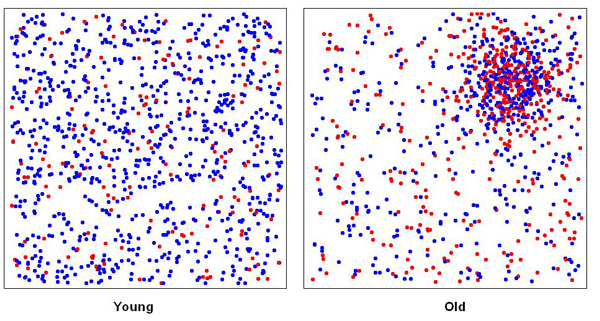
**Point map of synthetic data**. Locations of cases (red) and controls (blue) are shown stratified by a dichotomous variable (age). Disease odds are constant within strata, but four times higher in the old. Young are uniformly distributed; old are clustered in the northeast quadrant.

**Figure 2:**
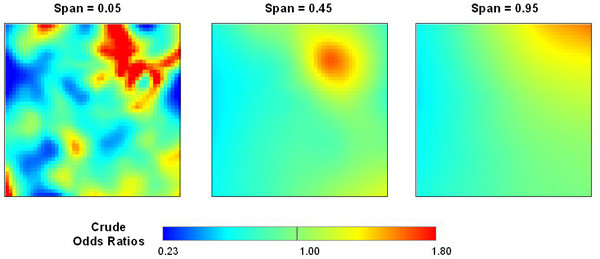
**Effect of span size on crude odds ratio map**. We use a generalized additive model to estimate smoothed log odds as a function of space and converted to odds ratios using the whole population as a reference. An optimal span of 0.55, chosen by minimizing the AIC, shows the correct underlying pattern, a single area of elevated disease in the northeast quadrant. Under-smoothing (left) or over-smoothing (right) distorts the pattern.

Figure 1 shows the distribution of the synthetic data by age and case-control status. Using the Akaike's Information Criterion (AIC), the optimal neighborhood size (i.e. percentage of the data) or span for the smooth term was 45 % of the synthetic data for the crude GAM model (omitting the age covariate). The resulting map shows an area of elevated odds ratios (OR) in the correct location, caused by the grouping of older subjects (Figure 2, center). An odds ratio of unity denotes areas with odds equal to the overall case-control ratio of 0.44. Crude odds ratios for the synthetic data ranged from 0.57 to 1.65, somewhat narrower from the expected range of 0.45 to 1.8.

To demonstrate the importance of span size, we also created crude maps using span sizes of 5% and 95% of the data. A span of only 5% produced a rough surface with one large cluster and various smaller clusters (Figure 2, left). The smaller clusters are due to random noise in the synthetic data, not an increase in disease risk, illustrating the danger of choosing too small a span size. Conversely, a span of 95% produced an over-smoothed surface (Figure 2, right). While the large span size identified only one area of increased risk, it was located further northeast.

**Figure 3:**
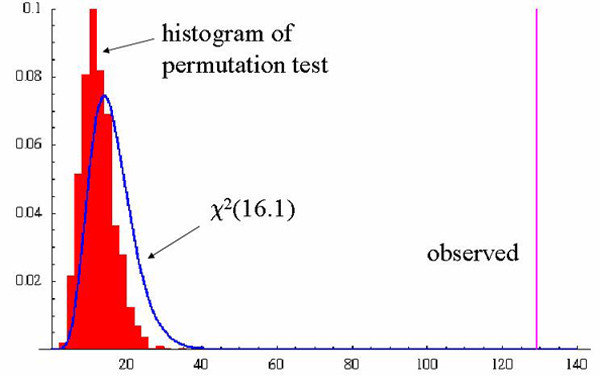
**Global test of location using deviance**. We test the global nullhypothesis of no association between location and disease status using the difference in deviance of models with and without the location term. We estimate the distribution of the statistic under the null hypothesis by permutation. The approximate chi-square distribution is also shown. The observed value of the deviance statistic is highly significant for the crude model (p < .0001) indicating that location is important, i.e., the crude map is not flat.

Returning to the crude map with the optimal span size, the two global permutation tests and the approximate chi-square test provided by S-Plus, testing the null hypothesis that case status does not depend on location (i.e. a flat surface), all resulted in very small p-values (p < 0.0001), indicating a highly significant association between location and disease status. As shown in Figure 3, the distribution of the deviance statistic estimated by permutations is shifted to the left compared with the chi-square distribution assumed by S-Plus. As the observed value of the statistic lies far to the right, both p-values are very small. However, if the observed statistic had been less extreme, the chi-square approximation would not have worked as well.

**Figure 4:**
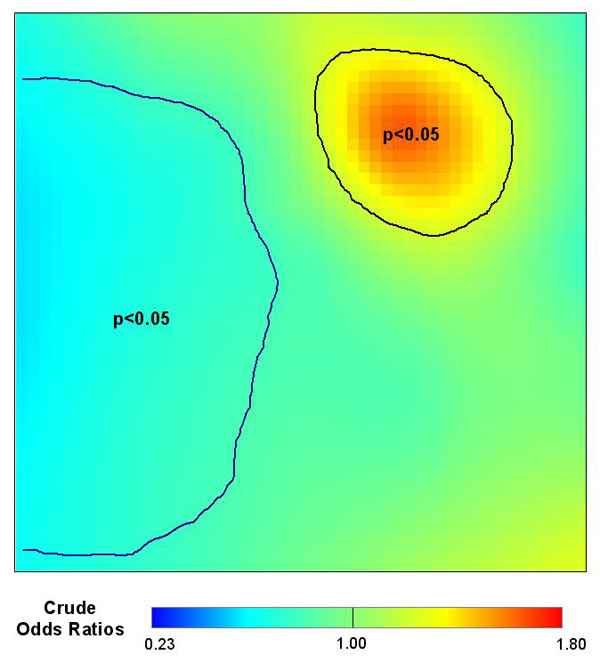
**Pointwise p-values**. We permuted the locations of subjects and reran the GAM model 999 times to estimate the distribution of log odds under the null hypothesis at each point. We define areas of significantly decreased odds ("cold spots") to include all points that rank in the lower 2.5% of the pointwise permutation distribution and areas of elevated odds ("hot spots") to include all points that rank in the upper 2.5% of the pointwise permutation distribution. We superimpose the 2.5% and 97.5% contour lines on the point estimate map. The slightly elevated, but non-significant, region in the lower right corner occurred due to chance.

With the global deviance test indicating that the map is unlikely to be flat, we next located areas of the map that exhibit unusually high or low disease odds. Figure 4 shows the results of the pointwise permutation tests. The 2.5% and 97.5% contours of the pointwise permutation distributions are drawn on the map of odds ratios. The region of significantly increased risk coincides with the area of elevated odds ratios. As the null hypothesis is a flat map with odds equal to the overall case-control ratio, there is also an area of significantly decreased risk.

**Figure 5:**
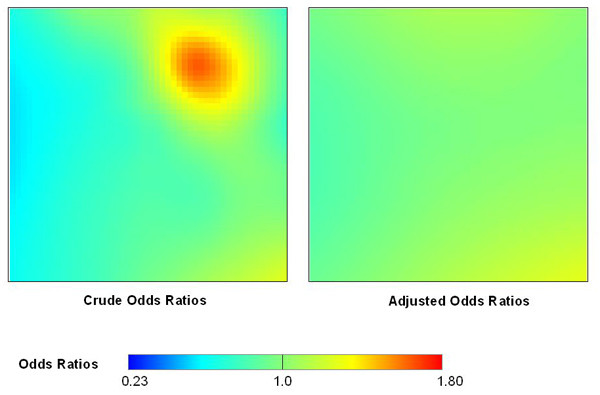
**The GAM model properly adjusts for a covariate**. The crude map ofthe synthetic data is elevated in the northeast quadrant due to spatial confounding, i.e., spatial clustering of the risk factor age. Adjustment for age produced a quite flat map, an expected result since we constructed the data assuming uniform disease odds within each stratum.

The map adjusted for age shows a flat surface compared with the crude map, demonstrating the presence of spatial confounding in the latter (Figure 5). A flat adjusted map is expected since the data were created assuming uniform disease odds within strata. The optimal span size chosen by the AIC was 95% of the data. There was no significant association between location and disease status in the adjusted map. The p-values for the global tests were 0.49 for the deviance statistic, 0.57 for the Kelsall and Diggle statistic, and 0.35 for the approximate chi-square statistic. The estimated odds ratio for the effect of age was 4.2 (95% CI = 3.2, 5.2), slightly higher than the value of 4 used to construct the simulated data.

**Figure 6:**
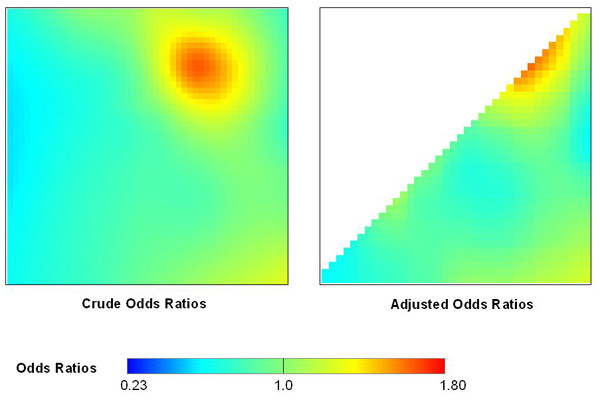
**Testing for edge effects**. GAMs can produce biased estimates near edges. We cut our data set in half diagonally and reran the model with the same span. The results are quite similar.

GAMs may exhibit edge effects, biased behavior at the edges of the data [5]. Loess, a locally-weighted regression smoother (see chapters 7 and 8 of [6]), uses a tri-cube weight function that down-weights points far from the target point, suggesting smaller edge effects than for nearest neighbor smoothers with the same span. To examine possible edge effects, we cut our data set diagonally in half and re-predicted the crude odds ratios with the optimal span of 45%. Figure 6 compares results from half the grid to that of the original grid. The self-imposed edge did not appear to affect the GAM's performance.

### Cape Cod data

The real data have two features quite different from the synthetic data. The population is concentrated mainly along the coast of the study area. In addition, the northeast interior of the study area has sparse population due to the presence of a military base.

**Figure 7:**
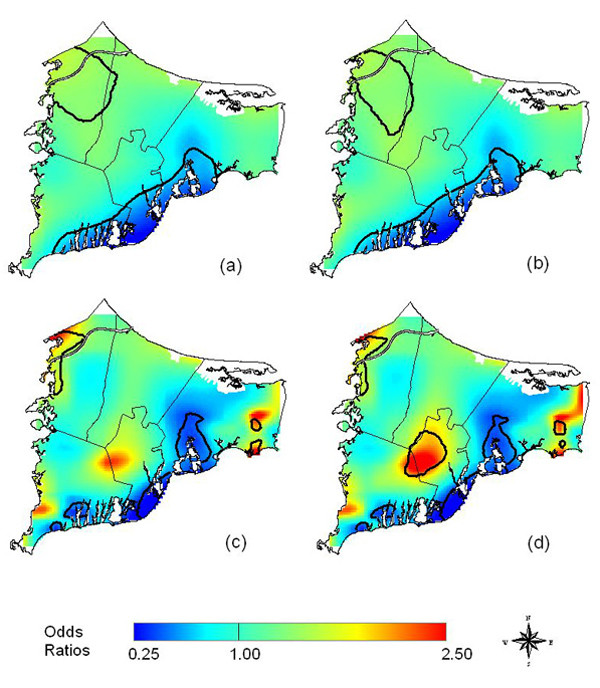
**Cape Cod Breast Cancer Data**. Twenty years oflatency. Odds ratios are relative to the whole study area. a) Crude, optimal span of 35%. b) Adjusted, optimal span of 35%. Adjusting for race makes little difference in the map at a span of 35%. c) Crude, span of 15%. A smaller span reveals hot spots not apparent at the larger span. d) Adjusted, span of 15%. Difference from the crude map indicates spatial confounding by race using the smaller span size.

Using the AIC curve, the optimal span for both the crude (omitting the race covariate) and adjusted GAM model in the 20-year latency analysis was35 % of the data. The two global permutation tests and the approximate chi-square test provided by S-Plus all resulted in very small p-values (deviance statistic = 0.002, Kelsall and Diggle statistic = 0.003, approximate chi-square statistic = 0.0008) for both the crude and adjusted maps, indicating a highly significant association between location and disease status. Adjusting for race slightly increased the odds ratios in the center of the study area as indicated by the subtle shift from green to yellow-green when comparing Figures 7a and 7b. The 2.5% and 97.5% contours of the pointwise permutation distributions are drawn on the maps of odds ratios.

**Figure 8:**
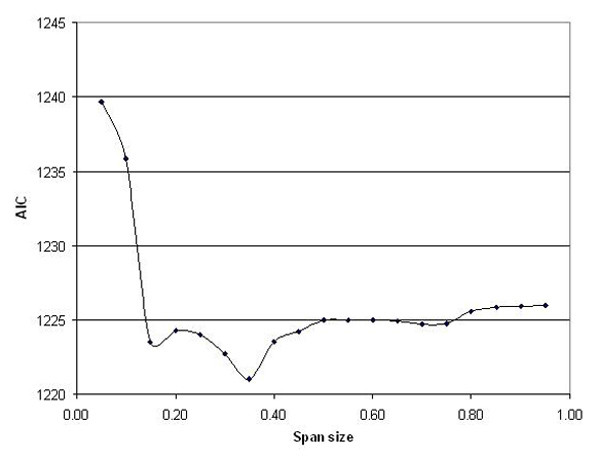
**Choosing an optimal span size**. Care must be used with automatic span selection procedures. Since the Cape Cod data showed both local and global minima for the AIC, searching from the left to find the first minimum underestimated the true optimum. More importantly, the AIC and other similar methods balance bias with variance, a goal not necessarily equivalent to locating important data features.

The AIC curve identified a second local minimum at a span of 15% (Figure 8). Using the smaller span size reveals various areas of elevated risk, or "hot spots", in the crude map that were not apparent using the larger span of 35% (Figures 7a, 7c). The global statistics resulted in very small p-values, once again indicating a highly significant association between location and disease status: 0.001 for the deviance statistic, 0.001 for the Kelsall and Diggle statistic, and 0.0001 for the approximate chi-square statistic. After adjusting for the covariate race, the hot spot in the center of the study region grows larger in geographic size and intensity and is now statistically significant as denoted by the contours of the pointwise permutation distributions (Figure 7c, 7d).

The effect of race is due to a large concentration of non-whites that were living in this area of upper Cape Cod (overall only 31 of 589 participants were non-white). Because non-white women have a lower risk of breast cancer, spatial confounding by race was partially masking the significance of location in the crude analysis. In the maps produced with the larger span size, it was necessary for the smoothing window to span across the sparsely populated northeast interior to the opposite coast in order to include the optimal number of residences, thus obscuring the effect of race.

## Discussion

Our goal is to develop practical methods for mapping population-based case-control and cohort studies. The method described here performs well for our synthetic data, reproducing important features of the data and adequately controlling the covariate. When applied to the Cape Cod data set, the method suggests spatial confounding and identifies statistically significant areas of increased and decreased odds ratios (For a more in-depth analysis of the Cape Cod data, see [7]).

### Comparison with other methods

In earlier papers, we constructed maps of case-control data using a nearest-neighbor smoother [8,9]. The span was based on the number of controls in a window rather than the number of subjects, so that every window calculated odds using the same denominator. While potentially useful, this non-standard smoother required special software and used *ad hoc *methods for span selection and hypothesis testing.

GAMs provide a unified statistical framework for smoothing binary and other kinds of outcome data, span selection, covariate adjustment, and hypothesis testing. Our current method differs in several ways from the earlier work of Kelsall and Diggle [10]. While both methods use generalized additive models, Kelsall and Diggle employed a kernel smoother, used cross-validation (CV) to select an optimal span size and tested the overall flatness of the map with a mean squared difference statistic. They used permutation to construct pointwise p-value surfaces and evaluate global statistics, but the permutations were based on reassigning case/control status based on fitted probabilities from a logistic regression based only on the covariates. They mapped log odds on a continuous scale. As kernel smoothers fix the size of neighborhoods based on distance, kernels may perform worse than loess when data density varies greatly [5], as population often does (for example, the population of Upper Cape Cod). While the mean squared difference and deviance statistics produced qualitatively similar p-values for our synthetic data, their relative power remains unknown. All differences between the two methods require further investigation.

### Limitations and future work

Despite the advances in mapping case-control data of the last decade, a number of issues remain. When evaluating the precision of a point estimate, many epidemiologists prefer confidence intervals to p-values [4]. Variability bands [11], a non-parametric counterpart to confidence intervals, may provide a useful technique. We are currently investigating this approach using bootstrapping.

We identified areas with significantly increased or decreased risk using pointwise hypothesis tests. By making these multiple comparisons we increase the likelihood of finding significant hot or cold spots by chance alone. Although we make no adjustment for multiplicity, we only conducted pointwise tests if the global deviance test indicated that the map was unlikely to be flat. The location of significant hot and cold spots should be considered as exploratory.

Both local and global minima of the AIC can exist. Starting at a small span size, the first minima in our Cape Cod data – and the one reported by S-Plus – occurs at 0.15, but the AIC is actually minimized at a span size of 0.35 (Figure 8). Should we use a single "optimal" span size? Automatic span selection methods such as the CV or AIC may be preferable to *ad hoc *procedures, but they should not be used blindly. Spans can be chosen to examine features at a particular scale if desired. Automatic span selection methods aim to balance the tradeoff between bias and variance, a goal not necessarily equivalent to detecting important features. We are currently exploring the application of "scale space" methods to our problem [12].

In the Cape Cod study, people live near coasts, increasing concern about edge effects. While we found little evidence for edge effects when applying our methods to synthetic data, additional work is clearly required.

Semiparametric studies of air pollution commonly employ GAMs: the effect of interest is modeled parametrically and several covariates are modeled with smooths. Dominici et al. [13,14] reported that S-Plus may produce a biased parametric regression coefficient with inflated standard error. Ramsay et al. [15,16] warned that stricter convergence criteria are not sufficient for eliminating these problems: concurvity, a nonparametric counterpart to multicollinearity, plays a role. We use our semiparametric model (1) differently: the effect of "exposure" (location) is modeled with a smooth while covariates are modeled parametrically. We assess the precision of the smooth with permutation tests, so inflation of software-provided standard errors is not an issue. As our parametric covariates are nuisance variables, bias of their coefficients or inflation of their standard errors is not a problem, provided that confounding is adequately controlled. Covariate control was adequate in our synthetic data, but additional work is needed, including more complicated covariates and multiple realizations of stochastic processes. As an additional check, we modeled the synthetic data using both default and more stringent convergence parameters; results were very similar (data not shown).

## Conclusion

In contrast to the well-developed methods for mapping area-based epidemiologic data, point data have received much less attention; adequate means of controlling covariates has been an important issue. GAMs provide a fairly simple solution to this problem. They provide a unified statistical framework for smoothing binary outcome data, controlling covariates, and testing hypotheses. They are a conceptually straightforward extension of familiar logistic regression and analyses can be performed with standard software packages. Although principally a tool for mapping, they provide both global and local tests of disease clustering [17]. When applied to population-based case-control and cohort studies with residential histories, a number of important questions may be addressed. Are apparent disease clusters due to (or masked by) spatial confounding? Does failure to take latency into account partially hide spatial patterns for diseases like cancer? GIS technology allows the overlaying of this next generation of disease maps with geographically-coded environmental and social information. Such comparisons may yield new exposure hypotheses.

## Methods

### Spatial analysis of area-based vs. point-based data

Methods for constructing maps of area-based disease data are well advanced [18,19]. In one of the simplest and most common applications, counts of cases in towns or other geographic units are linked to census data. Rates are then standardized directly or indirectly and mapped by area. Many of the statistical issues with such maps are well known. Multiple comparisons occur when many areas are tested for statistical significance using conventional criteria. The statistical stability of the rates depends on population sizes that typically vary greatly between areas. Numerous smoothing methods, such as empirical and fully Bayesian approaches have been developed in response [18,19]. Because of the limited number of individual-level covariates available from disease registries spatial confounding can occur. Poisson regression is one method for adjusting for additional covariates such as area-based measures of socioeconomic status, e.g., [20]. However, use of group-level covariates as proxies for individual-level variables may not control confounding, causing cross-level (ecologic) bias [21]. Other methods take into account residual spatial autocorrelation between areas, reviewed in [19].

Mapping of individual, point-based data presents different challenges. Point maps of cases alone are deceptive unless the underlying population is uniform. Mapping the locations of both cases and non-cases does not provide quantitative estimates of rates or adjust for covariates. Unless these data are aggregated back into areas, simple stratification and standardization methods are unappealing. A more fruitful approach models cases as an inhomogeneous Poisson process with intensity λ_1_(**x**) and controls with an inhomogeneous Poisson process with intensity λ_0_(**x**), where **x **is a vector describing location within the study area [20,22]. The density ratio method estimates the density of cases and the density of the controls using smoothing methods such as kernels. The ratio of the two densities estimates the spatial odds function. Alternatively, if locations of the complete population are available, the ratio of case density to control density estimates the spatial risk function. Unfortunately, the density ratio method provides no simple way to adjust for covariates [20]. However, we can consider the problem as a single Poisson process with intensity λ(**x**) = λ_1_(**x**) + λ_0_(**x**), labeled as to the case or control status of each person. Conditional on location, the odds of being a case equals the spatial odds function, which we can model via logistic regression

logit[*p*(**x**)] = α + **γ'z **+ *S*(**x**)     (1)

where the left-hand side is the log of the disease odds at location **x**, **α** is an intercept, **z **is a vector of covariates (individual and/or group-level), **γ** is a vector of usual regression parameters, and *S*(**x**) is spatial variation unexplained by the covariates [10,20].

Two statistical approaches have been proposed for modeling equation (1): a generalized linear mixed model formulation of kriging [23,24] and generalized additive models [10,25]. Both are promising but relatively untried methods in spatial epidemiology. For example, kernel-based GAMs have been used to map risks of lung cancer [10], biliary cirrhosis [26], and infant mortality [27].

### Generalized additive models

Generalized additive models (GAMs) describe the relationship between outcome and predictors without imposing specific parametric forms on the relationship [5]. GAMs provide a unified framework for mapping case-control data, allowing spatial smoothing of binary outcomes using a logit link while adjusting for covariates, selection of optimal degree of smoothing, and hypothesis testing.

To estimate maps of case-control data, we treat *S*(**x**) in equation (1) as a bivariate smoothing function *S*(*x*_1_,*x*_2_). Without the smooth function, *S*(*x*_1_,*x*_2_), the model reduces to an ordinary logistic regression on the covariates. Although one could in principle also model the covariates with smooths, we use the parametric form in order to decrease data requirements. Holding the covariates constant, a plot of the surface *S*(*x*_1_,*x*_2_) over all *x*_1_, *x*_2 _in the study area reveals the relationship between location and outcome, logit(*p*), adjusted for covariates. Omitting the covariates produces a crude (unadjusted) map. The S-Plus statistical package provides a GAM function to fit generalized additive models.

### Smoothing

Estimating the smooth *S*(*x*_1_,*x*_2_) requires two decisions: the type of smoother and the size of the neighborhood. As population densities often vary dramatically, we use loess, a locally-weighted regression smoother. Loess adapts the size of the neighborhood to the local density while maintaining the smoothness features of a kernel. This method defines neighborhoods based on the k-nearest subjects, weighting points within the neighborhood using a tricube distance function centered at a target point and decreasing to zero at the furthest neighbor [5]. S-Plus currently allows the use of loess or smoothing splines in GAM models, but only loess allows for bivariate smoothing, permitting simultaneous smoothing in two dimensions.

The amount of smoothing performed by loess depends on the size of the neighborhood of points. In general, small neighborhoods reduce bias but increase variance. Conversely, larger neighborhoods produce smoother surfaces resulting in increased bias and reduced variability. As the neighborhood increases in size, more data points receive non-zero weights and the loess smoother approaches a linear regression. Theoretical considerations use the bias and variance to provide several methods for choosing an optimal neighborhood size, also called bandwidth or span [5].

Kelsall and Diggle [10] used kernel smoothing to map disease, selecting an optimal degree of smoothing using weighted least squares cross-validation (CV). The CV minimizes an average squared predictive error criterion at every point *i *in the data set using the fitted value obtained by leaving the point *i *out of the sample. Minimizing this criterion over many possible spans is very computationally intensive because the model is fitted *n *times for each span choice, where *n *is the number of points in the data set. Wood [28] uses a less computationally intensive function to estimate smoothing parameters in generalized ridge regression with multiple penalties using generalized cross validation (mgcv). Krause and Tutz [29] provide a recent discussion of smoothing parameter selection in additive models. We choose an optimal span by minimizing the Akaike's Information Criterion or AIC [5]. Due to the lengthy computational time involved with CV and the availability of the AIC in S-Plus, AIC is commonly used as a method for automated selection of the optimal span size [30]. It approximates the deviance-based cross validation using the average deviance of a model penalized by the number of degrees of freedom. Both local and global minima of the AIC can exist. Depending on the starting point and breadth of the search, the S-Plus automatic span selection function, *step.gam*, may choose a local minimum as optimal rather than the global minimum. To find a global minimum, we plot the AIC curve for a large range of span sizes.

We estimate the crude and adjusted log odds at each location on the grid using the S-Plus function *predict.gam*. As this function defines neighborhoods based on a combination of the data points and the grid, it can produce discrepancies from predictions based on the original data alone [6,31]. We therefore check all maps to look for potential discrepancies, but these have always been minor.

### Significance testing

We first test the null hypothesis that case status does not depend on location, i.e., *S*(*x*_1_,*x*_2_) is a flat surface. The GAM approach provides a straightforward statistic: the difference of the deviances of model (1) with and without the smoothing term *S*(*x*_1_,*x*_2_). S-Plus provides an approximate p-value for this test based on the assumption of a chi-square distribution for the difference in deviances. Because the chi-square assumption is only approximate for GAMs and may be biased [5], we estimate the distribution of the statistic under the null hypothesis using a permutation test. We condition on the number of cases and controls, preserving the relationship between case/control status and covariates, and randomly assign individuals to locations. We carry out 999 permutations of location in addition to the original. For each permutation, we run the GAM using the optimal span of the original data and compute the deviance statistic. We divide the rank of the observed value by 1000 to obtain a p-value.

We also compute the global statistic used by Kelsall and Diggle [10]



where *r*(*x*_1_,*x*_2_) is the estimated log odds at each location and  is the average taken over all observed points. P-values are computed via a permutation test as described earlier. Recent work by Fan [32] also addresses testing additive components of a GAM.

If the global deviance test indicates that the map is unlikely to be flat, we next want to locate areas of the map that exhibit unusually high or low disease odds. We examine pointwise departures from the null hypothesis of a flat surface using permutation tests. We obtain a distribution of the log odds at every point using the same set of permutations we use for calculating the global statistics. We define areas of significantly decreased odds ("cold spots") to include all points that rank in the lower 2.5% of the pointwise permutation distributions and areas of elevated odds ("hot spots") to include all points that rank in the upper 2.5% of the pointwise permutation distributions.

### Calculating odds ratios

The GAM model yields two-dimensional arrays of smoothed, adjusted log odds. When controls are appropriately sampled from the population giving rise to the cases, the disease odds are proportional to the rate of disease. The proportionality constant – related to the sampling fraction – is generally not known. Assuming that the sampling fraction does not depend on location, differences of log odds between areas of the map are meaningful, as are the global tests of location and the pointwise p-value surface. But the absolute magnitudes of the log odds are not readily interpretable. Case-control studies usually remedy this situation by designating one group – typically the unexposed – as a reference group. Dividing the case-control ratio in an exposed group by the case-control ratio in the reference group yields an odds ratio (OR), an estimate of the ratio of the rates [4].

To simplify interpretation of the maps, we compute the odds ratio at every point using the whole study population as a reference. We divide the odds at each point by the "null" (aspatial) odds produced by equation (1) omitting the smoothing term. For crude models, this is equivalent to dividing by the ratio of the total number of cases to the total number of controls [8]. Thus, an OR of 1.5 at a location means that the rate of disease there is elevated 50% above the whole study population. We perform these calculations as a last step after all GAM estimation and statistical evaluation has been performed; one can therefore consider this step as a kind of normalization.

### Mapping

To obtain a map, we first create a rectangular grid based on the minimum and maximum latitude and longitude of the data set; the S-Plus GAM function does not predict outside of this range for a bivariate smooth. We map the grid of estimated odds ratios using ArcView. We clip grid points lying outside the outline map of the study area as well as areas where people cannot live, e.g., wildlife refuges. We use a continuous color scale to indicate the magnitude of the odds ratio [8], avoiding the need to assign the category breakpoints needed by a categorized choropleth map. After choosing a range of odds ratios to map, we assign dark blue to the minimum and dark red to the maximum. We display the odds ratios using a linear scale; although a log scale is a good option, it may be more difficult for many people to interpret. We map results using a divergent dark blue to dark red continuous (unclassified) color scale. Divergent scales are an effective way to communicate deviations of a map from flatness both higher and lower than unity (For a useful discussion of color schemes, see [33]). Blue and red are commonly associated with cold and hot, aiding interpretability of areas with decreased or increased disease odds. We denote areas of significantly decreased or increased disease odds by superimposing the 2.5% and 97.5% contours of the p-value surface on the map of point-estimates.

### Synthetic data

We generated synthetic data (n = 2000) to illustrate the method. Each datum had several elements: location on a unit square centered at zero, a dichotomous outcome, and a dichotomous covariate denoting "young" or "old". We generated 1000 locations for the young subjects using two uniform random variables centered at zero. For old subjects, we generated 500 locations using two uniform random variables centered at zero and 500 locations using two normal random variables each with mean 0.25 and variance 1/10^th ^that of the uniform distribution. We randomly assigned disease status assuming 1:5 odds of being a case for the young and four times this value for the old. These synthetic data were generated so that young are uniformly distributed, old are clustered, and the odds of disease are constant within each strata. The age covariate is thus both a risk factor and spatially clustered, i.e., a crude map should show spatial confounding, but a properly adjusted map should be flat.

In judging whether a variable is a confounder, epidemiologists typically compare the magnitude of adjusted and crude point estimates [4]. We analyzed the synthetic data using the model

logit[*p*(*x*_1_,*x*_2_)] = α + γz + *S*(*x*_1_,*x*_2_)     (2)

with and without the covariate term *z*, an indicator variable for young or old. We then visually compared the adjusted and crude maps of the synthetic data. To distinguish between changes due to adjustment and changes due to span, we created adjusted maps with two different spans: one optimal for the adjusted map, the other optimal for the crude map.

### Cape Cod data

To illustrate these methods using real data, we investigated the association between residence and breast cancer on Upper Cape Cod, Massachusetts (USA) using data from population-based case-control studies [34,35]. The Massachusetts Cancer Registry was used to identify incident breast cancer cases diagnosed from 1983–1993. Participants were restricted to permanent residents of the upper Cape region with complete residential histories. Controls were chosen to represent the underlying population that gave rise to the cases, i.e., permanent residents of the same towns during the same time period. Controls were frequency matched to cases on age, gender, and vital status.

Participants or their next-of-kin completed an extensive interview, providing information on demographics (age, sex, marital status, and education), a forty-year residential history, and potential confounders. "Index years" were randomly assigned to controls in a distribution similar to that of diagnosis years for cases. We used index years to estimate length and time of environmental exposure for controls in a fashion comparable to that of cases. See earlier papers [34,35] for a detailed description of the methods used to define the study population. The Institutional Review Board of Boston University Medical Center approved the research.

All residential addresses reported by participants in the upper Cape Cod area over the forty-year period prior to the diagnosis or index year were eligible for spatial analysis. Some participants lived at more than one location on Cape Cod or lived more than once at a single location if they moved away and later returned. We excluded all addresses where residency time began after diagnosis date for cases or index date for controls. Cancers initiated by exposure to environmental carcinogens typically take more than a decade to develop. We therefore performed a twenty-year latency analysis by restricting inclusion to the residences occupied by participants at least twenty years prior to the diagnosis or index year (Residences within the twenty year window were excluded because geographical location within that window was assumed not relevant to outcome). The breast cancer 20-yr latency data set included 248 cases representing 391 residential locations and 341 controls representing 509 residential locations.

For the purpose of illustrating our methods with real data, weanalyzedthe breast cancerdata usingonly one covariate term, an indicatorvariable for race, and one latency assumption (20 years). We assessed confounding by visually comparing the adjusted and crude mapsof the breast data. We used the AIC curve to identify the optimal span. In order to make maps visually comparable, we mapped all results using the same dark blue to dark red continuous color scale representing odds ratios ranging from 0.25 to 2.50, where odds ratios near unity appear as a light green. See Vieira et al. [7] for a thorough analysis and discussion of the complete data.

### Computation

We used S-Plus  for GAM estimation and significance testing, although R may provide a useful alternative [36]. We generated maps with ArcView [37]. Computations were performed on a Dell Dimension 8100 computer with a Pentium 4 processor. The time required for running a GAM was on the order of seconds. Program code and synthetic data are available on request.

## Abbreviations

AIC, Akaike's Information Criterion; GAM, generalized additive model; GIS, geographical information systems; OR, odds ratio

## Competing interests

The author(s) declare they have no competing interests.

## Authors' contributions

TW directed the study, drafted the manuscript, and collaborated on all analytical and editorial decisions. VV conducted the spatial analyses and provide analytical and editorial support. JW provided statistical support and consulted on analytical and editorial issues. AA provided the data and assisted in epidemiologic analysis and editing. All authors read and approved the final manuscript.
